# Workspace Analysis and Optimization of 3-PUU Parallel Mechanism in Medicine Base on Genetic Algorithm

**DOI:** 10.2174/1874120701509010214

**Published:** 2015-08-31

**Authors:** Yongchao Hou, Yang Zhao

**Affiliations:** 1 Department of Mathematics, Chaohu University, Hefei, Anhui, 238000, China; 2 Department of Electronic and Information Technology, Jiangmen Polytechnic, Jiangmen 529090, China

**Keywords:** Genetic algorithm, kinematics, limit search method, optimization analysis, parallel mechanism, workspace

## Abstract

A novel 3-PUU parallel robot was put forward, on which kinematic analysis was conducted to obtain its inverse kinematics solution, and on this basis, the limitations of the sliding pair and the Hooke joint on the workspace were analyzed. Moreover, the workspace was solved through the three dimensional limit search method, and then optimization analysis was performed on the workspace of this parallel robot, which laid the foundations for the configuration design and further analysis of the parallel mechanism, with the result indicated that this type of robot was equipped with promising application prospect. In addition that, the workspace after optimization can meet more requirements of patients.

## 1. INTRODUCTION

Compared with traditional serial robot, the parallel one, with large stiffness, superior carrying capacity, minor error and high accuracy, etc., has recently become a research hotspot in academia and been extensively applied in automobile, aviation, medical machinery and other fields, which has become an important trend in the development of parallel kinematic machines [1]. The parallel mechanism can check the body of patients and carry out Chest X-rays which is shown in Fig. (**1**). The parallel mechanism has numerous types, however, relative to multiple degrees of freedom (DOF), the lower-mobility parallel mechanism is simply structured with low cost and favorable flexibility, etc., which has more practical application value consequently, and various 3-DOFs parallel mechanisms have arisen. Na L [2] proposed a 3-PRPU three translational DOFs parallel robot, whose kinematics, Jacobian matrix and workspace were thoroughly analyzed; Yanwei Z [3] analyzed the kinematic performances of the spatial three-rotational-DOFs parallel mechanism; Lei W [4] put forward a three-DOFs parallel-legged hexapod bionic robot with promising medical application prospect; Cao [5] researched into the workspace and singularity of 3/3-RRRS parallel mechanism; Haipeng L [6]conducted optimization analysis on the workspace of the vertically-driven 3-PUU parallel mechanism based on Monte Carlo method.

The workspace, i.e. the working area of the parallel robot, serves as a significant index to evaluate the parallel machine performance. In this paper, on the basis of the inverse kinematics solution of 3-PUU parallel robot, the three-dimensional spatial model and the cross-sectional view of the robot were plotted with the employment of the visualization function of Matlab, and optimization analysis was 

performed. It was demonstrated that the optimization analysis was indispensible. The result can improve the workspace to meet more requirements of the patients.

## 2. ．KİNEMATİC ANALYSİS OF 3-PUU PARALLEL ROBOT

The structure diagram of 3-PUU parallel mechanism is shown in Fig. (**2**). Construct the basic coordinate system O−XYZ on the fixed platform, with X axis parallel to $OB_{1}$, Z axis vertical up to the fixed platform, and V axis given according to the right-hand rule, wherein the position vector of the slider was S_i_. Similarly, construct the mobile coordinate system P−UVW on the mobile platform, with *U* axis parallel to $PA_{1}$, *W* axis vertical up to the mobile platform, and V axis given according to the right-hand rule. Let the sides of the fixed platform construct an equilateral triangle, whose circumscribed circle radius was r_b_, and the radius of the mobile platform was r_a_.

Under the mobile coordinate system P−UVW, the position vector of Point A_i_ was:

(1) AP1=ra[100]T

AP2=ra[cos(2π/3)sin(2π/3)0]T

AP3=ra[cos(4π/3)sin(4π/3)0]T

Under the basic coordinate system O−XYZ, the position vector of Point S_i_ of the slider was:

(2) SO1=s1[100]TSO2=s2[cos(2π/3)sin(2π/3)0]TSO3=s3[cos(4π/3)sin(4π/3)0]T

The position of the mobile coordinate system relative to the basic one could be denoted by P as:

(3) $$P={\left[\begin{array}{ccc} x & y & z \end{array}\right]}^{T}$$

For the mechanism was equipped with three translational DOFs, there was no relative rotation between the mobile coordinate system and the basic one, consequently, the position vector of the mobile coordinate system under the basic one could be expressed as

(4) AOi=APi+P(i=1,2,3)

The inverse solution of the parallel mechanism could be denoted by the constraint equation of the fixed-length bar:

(5) L=‖AOi−SOi‖

Where in L is the fixed-length bar; and through the equation above, we could obtain

(6) $${\left(x+r_{a}-s_{1}\right)}^{2}+y^{2}+z^{2}=L^{2}$$

${\left(x-r_{a}/2+s_{{}_{2}}/2\right)}^{2}+{\left(y+\sqrt{3}r_{a}/2-\sqrt{3}s_{2}/2\right)}^{2}+z^{2}=L^{2}$

${\left(x-r_{a}/2+s_{3}/2\right)}^{2}+{\left(y-\sqrt{3}r_{a}/2+\sqrt{3}s_{3}/2\right)}^{2}+z^{2}=L^{2}$

If the basic dimensions of the mechanism and the position of the mobile platform are known, the displacement of the sliding pair could be solved through Eq.(6), wherein s_i_ is taken as positive.

## 3. WORKSPACE ANALYSİS OF 3-PUU PARALLEL ROBOT

The locations that the end reference points of the parallel robot could reach will constitute a set, i.e. the so-called workspace of the robot, whose size represents its activity range, which is a significant kinematic index to evaluate its service capacity and therefore exhibits a significant importance in the workspace analysis of the parallel robot [7].

### 3.1 Main Influencing Factors on the Parallel Robot Workspace 

During the actual operation, there principally exist the following factors influencing the parallel robot workspace [8-11]:

(1)        Sliding pair limitation: due to the limitation from the mechanical structure, the sliding pair was restricted to a certain range. When the mobile platform moves, the restraint to the slider stroke will not exceed the maximum measurement range, i.e.$s_{i\min}\leq s_{i}\leq s_{i\max}$, wherein $s_{i\max}and\text{​}s_{i\min}$are the maximum and the minimum measurement limits, respectively.

(2)        Rotation angle limitation of Hooke joint: the mobile platform of the parallel mechanism was connected to the fixed-length bar through the Hooke joint, while the slider, also connected to the fixed-length bar through the Hooke joint, was laid on the slide rail of the base. Generally, the wider the rotation angle range, the bigger the workspace of the mechanism. Nevertheless, given the limitations from the mechanical structure itself, the rotation angle range of the Hooke joint is finite with fixed value during actual operation. As a consequence, let the included angles between the fixed-length bar and the platform of 3-PUU parallel mechanism be $U_{bi}\;and\text{​}{\;U}_{ai}$, respectively.

(7) $$U_{bi}=\arccos(eb\cdot L_{i}/\left|eb\right|\left|L_{i}\right|)\leq U_{\max}$$

$U_{ai}=\arccos(ea\cdot L_{i}/\left|ea\right|\left|L_{i}\right|)\leq U_{\max}$

Wherein ea and eb are the normal vectors of the mobile and fixed platforms, respectively; L_i_ is the direction vector of the fixed-length bar, and $U_{\max}$ is the maximum rotation angle of the Hooke joint.

(3) Interference between bars: the swinging angle of any drive bar relative to either platform of the two is bigger than the maximum allowable one of the Hooke joint or spherical hinge at the articulation joint of the drive bar and the platform; and the telescoping range of the drive bar is bigger than the maximum allowable stroke of its sliding pair; thus the two drive bars interfere with each other.

(4) Singular configurations: singularity is an unavoidable phenomenon for all the machines, which is inherent to multiple spatial mechanisms and exhibits a big influence on its operation performance. When the mechanism is at certain specific configuration, its Jacobian matrix is singular with the determinant of 0, thus the mechanism is singular. During the kinematic analysis, the singular configuration of the mechanism is significantly important. Furthermore, for any spatial mechanism, the singular points or areas must be rejected from the workspace, and the singular location shall be avoided as much as possible during the working process.

### 3.2 Monte Carlo Method

In this paper, the Monte Carlo method was to be employed for the workspace analysis of 3-PUU parallel robot, detailed as follows. The Monte Carlo method is also called the extreme limit boundary searching method, which is conducted based on the inverse solution of the kinematic location of the parallel mechanism, with the principle as following: give a range containing all the possible workspaces of the parallel robot, wherein a large quantity of random points will be generated, and every point will be judged whether to be in the workspace or not. If it meets the constraint condition, it belongs to the workspace, if not, it shall be rejected. All the feasible points will constitute the workspace of the mechanism. And the one from the qualified to the unqualified is just the boundary point of the workspace, and the line constituted is the boundary line [12-14].

The detailed steps of the limit boundary searching method for the workspace were listed as following:

(1) Estimate the approximate workspace range as per the detailed structural parameters of the parallel robot.

(2)        Segment the workspace into n subspaces, ΔZ in thickness, by the plane clusters parallel to X-Y plane, and let the subspace be a cylinder with the height of, subsequently, segment X-Y into minor subspaces with the thickness of $\Delta Z\;\mathrm{from}\;z_{\min}\;\mathrm{to}\;z_{\max}$, and search along axis z.

(3)        In each subspace, conduct a step-by-step search with the polar angle progressively increasing by Δγfrom 0 to 2π, and the polar radius by Δρ from 0 to ρ, subsequently solve the slider location of the sliding pair or the rotation angle of Hooke joint, when it jumps from the qualified inverse solution to the unqualified one, or from the unqualified to the qualified, this point is just the boundary point of the workspace, and the set obtained is the workspace boundary line, as shown in Fig. (**3**).

(4) Search out the workspace boundary points of all the subspaces, which could produce the enveloping surface of the workspace, and the points in the curved surface constitute the workspace of the parallel mechanism.

The structural parameters of 3-PUU parallel mechanism were set as: the radiuses of the mobile and the fixed platforms were ra=90mm  and rb=700mm, respectively, the length of the fixed-length bar was l=300mm, the activity range of the slider was 50−500mm, and the maximum rotation angle of the Hooke joint was π/3. And then with Point P of the mobile platform as the reference point, the method above was employed to analyze the workspace that the reference points could reach and begin searching[15-16]. Within the range of −200≤x≤ 200  , −200≤y≤200    and    280≤z≤400, the search step length of each variable was set to be 4, subsequently, the position points obtained were substituted into the inverse equation, with the unqualified points rejected, finally, through the employment of Matlab, the three-dimensional stereoscopic graph of the workspace of this parallel robot were plotted as shown in Fig. (**4**).

It could be seen from 3-PUU in Fig. (**4**) that the workspace of this parallel mechanism was relatively big without any cavity, indicating that the workspace is applicable to the operation in a larger workspace. 

## 4. OPTİMİZATİON DESİGN OF PARALLEL MECHANİSM BASED ON GA

The workspace of 3-PUU parallel mechanism will be directly influenced by the structural parameters [17-19]. As a consequence, it is necessary to optimize the structural parameters, so as to maximize and optimize its workspace.

### 4.1 Optimization Model Establishment

The structural parameters of 3-PUU parallel mechanism include the radius $r_{a}$ of the mobile platform, the radius $r_{b}$ of the fixed one and the length l of the fixed-length bar. And the constraint condition factors include the rotation angle $U_{i}$ of the Hooke joint and the translational displacement $s_{i}$ of the drive pair. Take the ratio of the feasible point number of the workspace and the search point number of the whole space as the target function of the workspace, i.e.

(8) $$\eta =\frac{N_{feasible}}{N_{total}}$$

The optimized mathematical model was $\eta_{\max}$ and the constraints were showed as the followed:

(9) $$\text{S}.t.\left\{ \begin{array}{c} 70\leq r_{a}\leq 100\\ 650\leq r_{b}\leq 750\\ 280\leq l\leq 350\\ U_{i}\leq \pi /3\\ 100\leq s_{i}\leq 500 \end{array}\right.$$

### 4.2 Optimization Algorithm Selection

Genetic algorithm is a kind of machine learning technique, which relies on evolutionary theory [20-22]. Unlike traditional optimization methods, genetic algorithm is a colony optimization technique. In the algorithm, individuals are often decoded by binary system. Individuals need to be decoded during fitness calculation. Fitness is an index for evaluating the advantage and disadvantage of a solution. According to Darwin’s evolutionary theory, individuals with high fitness are more likely to be selected to generate the next generation. 

Based on the selected individuals, the next generation is generated by genetic operators. Each of the individual conducts cross over and mutation with another selected individual according to certain probability. The generated individuals become the candidate solutions for the next generation. This process is repeated for many generations to enable the population to evolve constantly, thus acquiring the solution of the optimization problem, as shown in Fig. (**5**). The genetic algorithm is characterized by: 1). Preserving a small number of elites in the population to the next generation. 2). Automatically determining the size of the population according to the defined optimization problem. 3). Providing 3 categories of cross over operations, i.e., single-point cross over, two-points cross over and uniform cross over. 4). Mutating the individuals according to certain probability. 

And the parameter configuration of GA was as follows:

Scale of sub-population: 10

Scale of total group：200

Total evolving algebra: 100

Crossover rate：0.7 

Mutation probability: 0.01

Elite individual: 1

### 4.3 Optimization Result Analysis

The genetic algorithm toolbox of Matlab was employed to conduct optimization analysis of this mechanism, with the optimal parameters obtained as$r_{a}=98,r_{b}=720\;and\;l=320$. And then the optimized mechanism parameters were utilized for the workspace graphing as shown in Fig. (**6**).

It can be seen from Fig. (**4**) and Fig. (**6**) that the workspace was obviously enlarged, which demonstrated the necessity of the optimization analysis of the mechanism. In addition that, the workspace after optimization can meet more requirements of patients.

## CONCLUSİON

In this paper, with the novel three-translational-DOFs parallel mechanism 3-PUU as the research object, the inverse kinematics solution equation was established; subsequently, on the kinematic basis, the workspace of the mechanism was analyzed, wherein the constraints of the drive, the rotation angle, the singularity and the interference were taken into consideration, with the limit boundary searching method of Monte Carlo employed, the workspace was eventually solved; Moreover, the visualization graphing of the workspace was performed with the aid of Matlab, and so as to guarantee the normal operation of the mechanism, the working range shall be within the permissible workspace. Furthermore, the genetic algorithm (GA) was utilized to conduct optimization of the mechanism parameters, with the result indicating that the workspace was obviously enlarged. Overall, the workspace of the parallel mechanism under research laid the foundations for the actual engineering applications.

## Figures and Tables

**Fig. (1):**
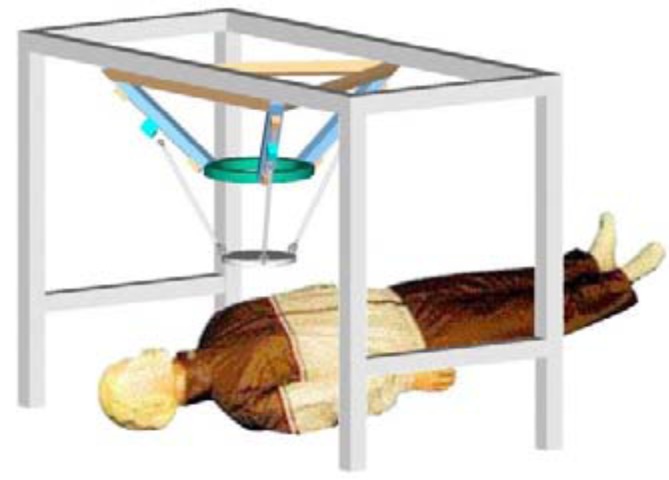
The application in medicine of 3-PUU.

**Fig. (2):**
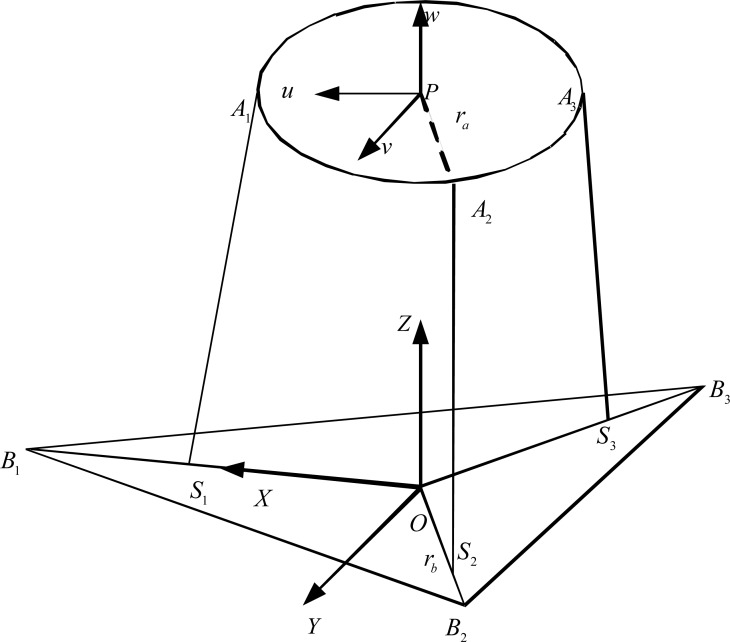
Structure diagram of 3-PUU parallel mechanism.

**Fig. (3):**
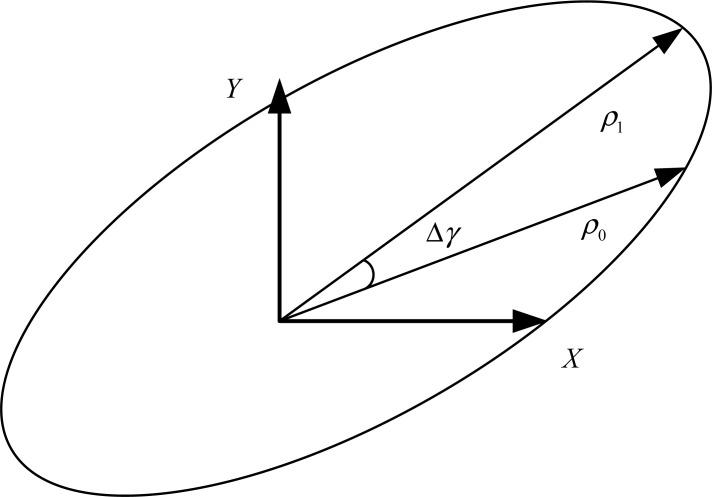
Cross-sectional view of workspace.

**Fig. (4):**
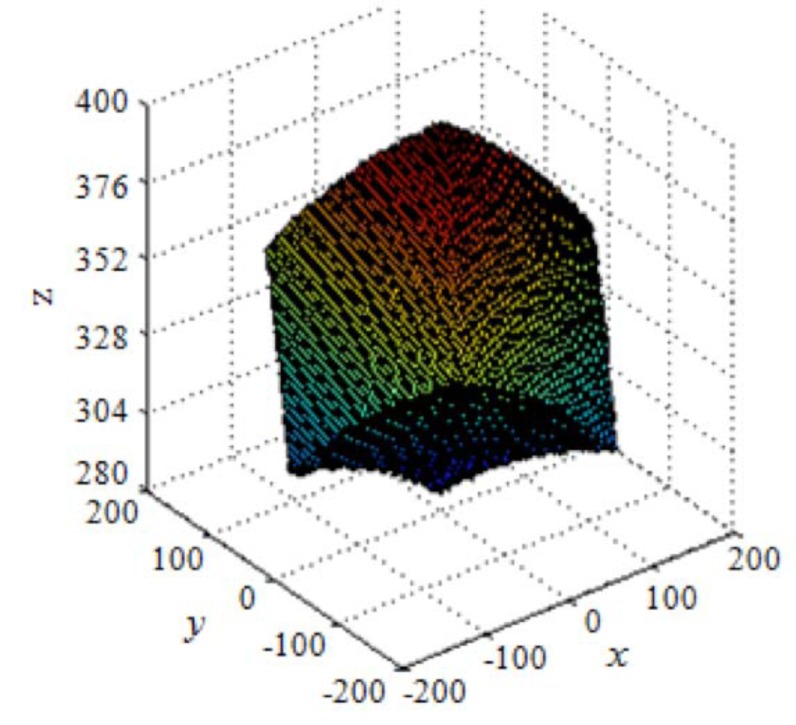
Three-dimensional spatial graph of 3-PUU parallel mechanism.

**Fig. (5):**
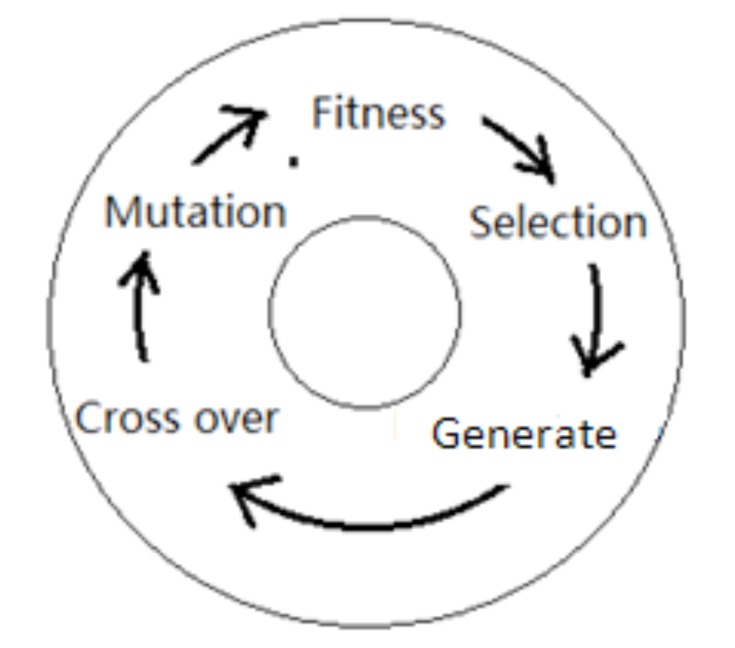
Genetic algorithm.

**Fig. (6):**
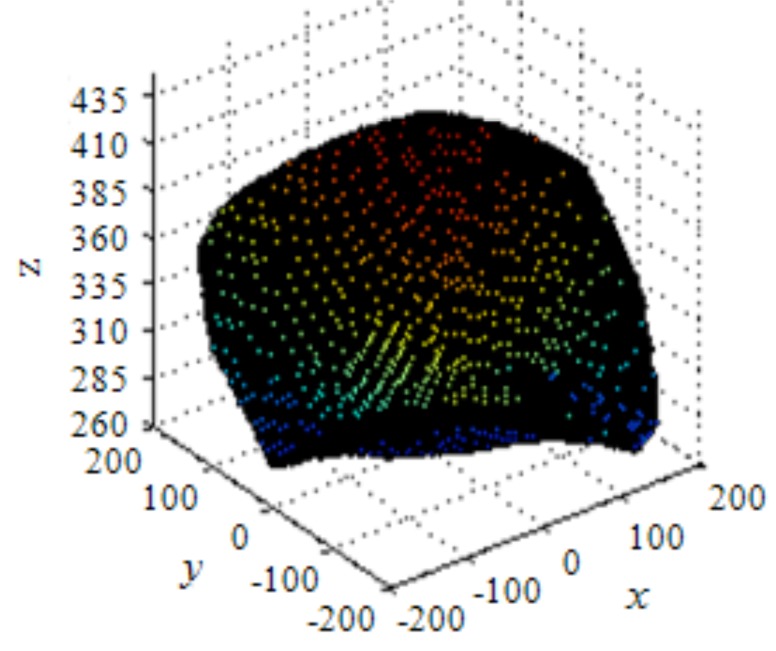
Three-dimensional space after optimization.
